# Patient and public involvement in dementia research in the European Union: a scoping review

**DOI:** 10.1186/s12877-019-1217-9

**Published:** 2019-08-14

**Authors:** Jahanara Miah, Piers Dawes, Steven Edwards, Iracema Leroi, Bella Starling, Suzanne Parsons

**Affiliations:** 1 Division of Neuroscience and Experimental Psychology, University of Manchester, Jean McFarlane Building, Oxford Road, Manchester, M13 9PL UK; 2 Manchester Centre for Audiology and Deafness (ManCAD), Manchester Academic Health Science Centre, University of Manchester, Oxford Road, Manchester, M13 9PL UK; 3 Public Programmes Team, Research and Innovation Division, Manchester University NHS Foundation Trust and The University of Manchester, 29 Grafton Street, Manchester, M13 9WU UK

**Keywords:** Patient and public involvement, Dementia research, Care partners, European Union

## Abstract

**Background:**

Internationally, there is a drive to involve patients and the public in health research, due to recognition that patient and public involvement (PPI) may increase the impact and relevance of health research. This scoping review describes the extent and nature of PPI in dementia research in the European Union (EU) and summarises: (i) how PPI is carried out; and (ii) the impact of PPI on people living with dementia and the public, researchers, and the research process.

**Methods:**

Relevant studies were identified by searches in electronic reference databases and then filtered by two reviewers independently. Eligibility criteria for included studies were: (i) people living with dementia and/or care partners; (ii) PPI activity in dementia research conducted in the European Union (EU); and (iii) published between 2000 and 2018. An adapted version of the Guidance for Reporting Involvement of Patients and the Public (GRIPP2 SF) was used to collate the data. There was no language restriction other than the abstract needed to be available in English.

**Results:**

We found 19 studies from the UK and one from the Netherlands meeting inclusion criteria. No studies from other EU countries met inclusion criteria. Studies reported various methods of PPI including workshops, drop-in sessions, meetings, consensus conference, reader consultation and participatory approach. The reported aims of PPI included identifying and prioritising research questions (*n* = 4), research design (*n* = 5), undertaking and managing research (*n* = 8), and data analysis and interpretation (*n* = 3). All PPI related to design and implementation of non-pharmacological studies. One study described two pharmacological studies as case studies incorporating PPI. Seventeen studies reported anecdotal impacts of PPI.

**Conclusions:**

Further development of PPI in dementia research in the EU and in pharmacological dementia research is required. Given the wide range of objectives of PPI in dementia research, PPI methods should be flexible and appropriate for the research context. Researchers should also formally evaluate and report the impacts of PPI for researchers, patients and the general public using good quality research designs to foster development of the field and enable the benefits and challenges of PPI to be better understood.

**Trial registration:**

PROSPERO 2017: CRD42017053260.

**Electronic supplementary material:**

The online version of this article (10.1186/s12877-019-1217-9) contains supplementary material, which is available to authorized users.

## Background

Patient and Public Involvement or PPI in research is described as “doing research with or by the public, rather than to, about, or for them” [1]. PPI involves recognising the importance of patients and public’s viewpoints and concerns, and that the view of patients and the public may differ from those of researchers [1].

PPI in health research has become increasingly common due to the growing recognition that it may increase the relevance and utility of research outputs to patients and the general public and in turn make the research more cost-effective [2]. It is also considered to be ethically desirable to involve people in research that pertains to them [2, 3]. PPI may also increase the effectiveness of research by making recruitment materials easy to understand, advising on appropriate recruitment strategies, and suggesting implementation and dissemination strategies [4, 5].

Across Europe, healthcare policies recognise the importance of PPI in research [6, 7], and the European Union Clinical Trials Regulation (due to come into effect in 2019) recommends PPI as a quality standard for clinical trial design [8]. European charities and patient groups including the European Lung Foundation, the European Patient Ambassador Programme, the European Patient Forum, and the European Patients’ Academy on Therapeutic Innovation, also advocate PPI in research to ensure patients and the public can influence the development and delivery of health research [9].

Following the growing interest in PPI in health research, within dementia research specifically, there is increasing interest and awareness of the need for PPI. Alzheimer Europe [10, 11] has recommended involving people living with dementia in research, and national governments and charities have emphasised that people living with dementia and their care partners have the right to contribute to research [12, 13]. To help us understand the extent of PPI within dementia research in Europe, we undertook a scoping review to map out this area which has not been previously reviewed.

This review summarises: (i) how PPI with people living with dementia is being carried out; and (ii) the impact of PPI on people living with dementia and the public, dementia researchers, and the research process within the European Union. The focus is on studies in European Union due to unique research, medical and social care arrangements within the European context.

## Methods

We followed the guidance on Preferred Reporting Items for Systematic reviews and Meta-Analyses extension for Scoping Reviews (PRISMA-ScR) Checklist for this scoping review [14] (See Additional file 1). For the purposes of this review, PPI is defined as involvement in research being carried out ‘with’ or ‘by’ members of the public rather than ‘to’, ‘about’ or ‘for’ them [1], the term ‘care partner’ is denoted as a spouse, family member or professional caring for a person living with dementia. Additionally, for the purpose of summarising the data, the term ‘impact’ refers to the outcome as a result of the changes made from PPI input in the papers identified for this review (Table 1).

**Table 1:** Possible outcomes pertaining to patient and public involvement in research [2]

Patient and public involved	
• New skills and knowledge • Personal development • Support and friendship • Enjoyment and satisfaction • Financial rewards	
Researchers	
• A better knowledge and understanding of the community • Enjoyment and satisfaction • Career benefits • Challenges to beliefs and attitudes	
Research process	
• Identifying topics for research • Shaping the research agenda • Reshape and clarify the research question • Improvements in the design of research tools • Research methods have worked in practice • Increased participation rates • Enhanced the quality of the data • Help engage the target audience, enhance the credibility of the findings	

### Patient and public involvement in the review

We involved public contributors in this review to help us understand what the review findings mean for people with dementia and care partners and to inform our learning from the review findings. Three public contributors to a dementia focused research advisory group (including one person with early onset of dementia and two care partners, all aged over 65 and based in the UK) was consulted via a face-to-face meeting to discuss their perspectives on the emerging findings from this scoping review.

The public contributors were informed about the purpose of the meeting, given an overview of what a literature review is, and what the aims of the review were. The group was then presented with a short version of Table 4 printed in large fonts. The PPI group was asked to comment on whether they agreed that PPI is important in dementia research, and what they thought about the different PPI methods identified in this review. We asked for the group’s views on what would be a good outcome if they were involved in dementia research and whether there would be any disadvantages of any of the approaches identified in the review.

### Research questions

This scoping review addressed the following questions in relation to PPI in dementia research:

1. How is PPI being carried out with people living with dementia within the European Union?
2. What are the reported impacts of PPI on people living with dementia and the public dementia researchers, and the research process within the European Union?

### Inclusion criteria

The population, intervention, comparison, outcomes and study design (PICOS) structure guided the study inclusion criteria for this review. Inclusion criteria were: (i) people living with dementia and/or care partners; (ii) PPI activity in dementia research conducted in the European Union (EU); and (iii) published between 2000 and 2018. The time period from 2000 to 2018 was selected to ensure that recent practice was included. There was no language restriction other than the abstract needed to be available in English. There were no restrictions on study design and grey literature was included. We excluded studies reporting public engagement activities which focused on dissemination of research [1]. Book reviews, opinion pieces, unpublished theses and literature reviews were also excluded.

### Search strategy

We used the term ‘patient and public involvement’ as a starting point to develop a search string and identified additional keywords that were used in articles to refer to PPI; this enabled us to build a free text search strategy for PPI (Table 2). We then combined the search string for PPI with the Medical Subject Heading (MeSH) keywords for ‘dementia’ (Table 2).

**Table 2:** Search terms

Search terms for ‘Patient and Public Involvement’	MeSH search terms for ‘Dementia’
Patient* involvement, public involvement, Patient* and public Involvement, involving Patient*s, user led, service user involvement, Patient* participation, patient* and public Voice, study partner, participatory research, Consumer involvement, citizen participation, Patient* and service user involvement, user Involvement	Dementia, Dementia with Lewy bod*, Lewy bod*, Mild cognitive impairment, MCI, AD, Alzheimer* disease, Memory loss, Huntington* disease, Primary progressive aphasia, Vascular dementia, Parkinson* disease, Frontotemporal dementia, Frontotemporal Lobar degeneration

*denotes truncation symbol

### Database searching

Bibliographic databases were searched from 2000 to 2018 (See Full Search Terms, Additional file 2). Search databases included MEDLINE and MEDLINE in Process (via Ovid and PubMed), EMBASE, CINAHL, InvoNET, Health Technology Assessment Database (DARE), Cochrane Library including the Cochrane Central Register of Controlled trials (CENTRAL), PsycINFO, BMJ Journals Online Collection, British Nursing Index, EBSCOhost Research Databases, OpenGrey and GreyNets, Web of Science and Google Scholar.

### Study selection

JM and SE independently reviewed the titles and abstracts against the eligibility criteria of 9203 studies. Full texts of potentially eligible studies were then manually filtered by JM and SE, a third reviewer (SP) was consulted for any disagreements and reconciled through discussion.

### Data extraction

The review protocol was used to develop a data extraction form (See Additional file 3). Form fields included study characteristics (author, country and year of the publication, study aims, study population, study design), the PPI term used, PPI approach, whether PPI impact was evaluated, how PPI was evaluated and evaluation findings.

### Collating and summarising data

Due to the wide variation in PPI approaches in the included studies and the variability of the quality of reporting of PPI, a descriptive approach was used to summarise the results of the review. This involved descriptive summary tables with headings based on the adapted Guidance for Reporting Involvement of Patients and the Public, short form version 2 (GRIPP2-SF) [15] guidelines (Table 3) to describe and summarise the data. Adaptation was made to the GRIPP2-SF [15] by adding the following additional fields (i) which EU countries the study had taken place (ii) the population involved and (iii) what PPI terms were used and how this was reflected in the study methodology. Additionally, because our review aimed to identify the impact of PPI, we added fields to capture whether studies incorporated systematic evaluation methodology describing how the impact of PPI was assessed on PPI members, researchers and on the research process and what the conclusions of the evaluation were.

**Table 3:** Adapted Guidance for Reporting Involvement of Patients and the Public 2, short form [15]

Section and topic	Item
1: Country^a^	EU country the PPI study was conducted
2: Aim	Report the aim of PPI
3: PPI term used^a^	Term used to describe PPI
4: Population^a^	Which patient or public population took part in PPI
5: Methods	Provide a clear description of the methods used for PPI
6: Study results	Report the results of PPI on the research process impact on researchers and PPI members, including both positive and negative outcomes
7: Discussion and conclusions	Comment on the extent to which PPI influenced the study overall
8: Reflections/critical perspective	Comment critically on the study, reflecting on the aspects of involvement that went well and those that did not
9: Evaluation methods^a^	Methods used to evaluate the impact of PPI on researchers, on patient and public involved, on the research process
10: Findings from evaluation^a^	The impact of PPI on researchers, on patient and public involved, on the research process

(^a^adapted sections)

We used the NIHR’s research process model [16] to describe which stage of the research process research-oriented PPI was focussed on, including; identifying and prioritising, design, undertaking/managing, analysing and interpreting, dissemination, implementation, monitoring and evaluation.

## Results

**Fig. 1:**
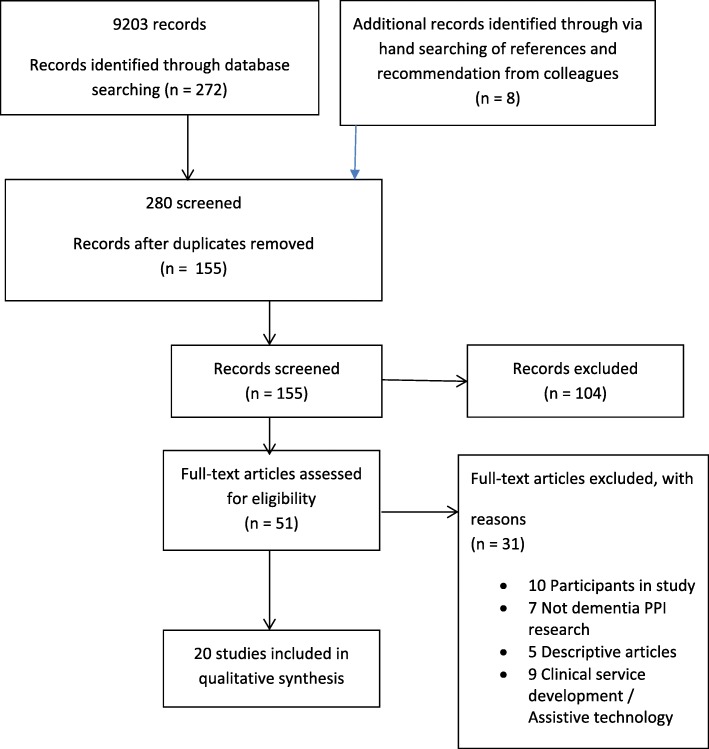
Flow of studies as per PRISMA flow diagram

9,203 studies were identified through database searching, eight were identified through other sources (hand searching of references and recommendation from colleagues), yielding 155 studies after removal of duplicates. Fifty-one full text papers were assessed, and twenty studies were included (Fig. 1). Thirty-one studies were excluded: ten were research studies with people living with dementia and care partners as participants; nine studies reported PPI in clinical service development/assistive technology; seven reported PPI but the PPI activity was not in relation to dementia research; five articles were commentaries citing dementia and PPI. Nineteen of the included studies were conducted in the UK and one was undertaken in the Netherlands. They were all published between 2005 to 2018 (see Table 4).

**Table 4:** Study Characteristics: Dementia research

NIHR Research Process	Reference	Country	Aim	PPI term used	Population	Methods	Study results Outcomes	Discussion and conclusions outcomes	Reflections/critical perspective	Evaluation method	Findings
Identifying & prioritising	Alzheimer’s Society (2013) [17]	UK	To bring together a wide range of organisations representing the views of people affected by dementia, practitioners and clinicians to collectively agree on priorities addressing the care, treatment, diagnosis and prevention of dementia	Priority setting partnership	PwD, carers, and health and social care professionals	• Consultation - survey • Workshop - Nominal Group technique	Questions were prioritised by PwD, carers, and health and social care professionals to inform the future of dementia research	NR	NR	NR	NR
Identifying & prioritising	Deane et al. (2014) [18]	UK	To encourage people with direct and personal experience of Parkinson's disease to work together to identify and prioritise the top 10 evidential uncertainties that impact on everyday clinical practice for the management of Parkinson's disease	Priority setting partnership	PwP, carers and former carers, family members and friends, healthcare and social care professionals	• Consultation - survey and • Consensus meeting/ workshop• Steering group	Identified the paucity of evidence currently available to address the everyday practicalities of managing a complex disease such as PD. These results will help funders identify future priorities for research that have greatest relevance to patients and the clinicians that treat them.	NR	NR	NR	NR
Identifying & prioritising	Poland et al. (2014) [19]	UK	Carers and people with dementia to identify key issues for developing carer-relevant research projects	Patient and public involvement workshop	PwD and carers	• Workshop using adapted focus group methodology	Discussions highlighted the need to understand why health care professionals might not spend time listening to patients and carers so as to address their medication related difficulties and concerns	Directly involving carers in specifying topics that matter to them, can greatly enhance the relevance and feasibility of research to develop effective interventions	Involving health professionals into dialogue with carers in an extended workshop may have brought more perspectives to demonstrate the extent of difference in their experience, understanding and weighting of medications issues.	NR	NR
Identifying & prioritising	Hassan et al. (2017) [20]	UK	To involve members of the public in discussions about the acceptability and feasibility of different devices and research designs to inform the development of a device pool, software platform and written guidance to support future studies	Patient and public involvement	PwD and cognitive impairments, carers and people without memory problems	• Workshops• Drop-in sessions • Meetings	Findings were used to develop a pool of devices for researchers, with computer software and written guidance to help plan, design and support studies. Lessons learned was the need to build in help with set-up and on-going technical support as part of future connected health dementia research studies, as well as support with operating the device itself	It was feasible to involve patients and the public and use their insights to shape the development of a sensing platform for dementia research. Seeking feedback from a range of potential user groups meant that their requirements are accounted for	NR	NR	NR
Design	Paterson et al. (2005) [21]	UK	User participation in a pilot study to inform the design of any subsequent large-scale trial	User participation	PwP	• Semi-structured interviews	Service users assisted the team to create relevant outcome measures and helped with refining the design of the trial by advising on how best to explain the intervention to participants, helped the research team in defining the inclusion criteria and also highlighting an issues; relating to the time of the day when the interview is conducted with PwP’s, as people’s symptoms alternate during the day and are affected by their medication. The service users recommended that researchers assist participants to complete any questionnaires as people with Parkinson’s have may find it stressful to talk about their illness, and have problems with concentration and their eyesight	Study confirms the benefits of involving users in the research process and makes recommendations concerning the design of any future randomised trial	NR	NR	NR
Design	Yates et al. (2015) [22]	UK	Service users’ involvement in the development of individual Cognitive Stimulation Therapy for dementia	User involvement	PwD and carers	• Semi-structured interviews • Focus groups	Informed the development of the second draft of the manual, sample materials presented were well received by both carers and people with dementia, valuable insight into the feasibility of the programme, identified potential barriers at an early stage	Yielded valuable insight into the needs of service users for the iCST programme, and the importance of mental stimulation, both from the point of view of carers and people with dementia	NR	NR	NR
Design	Brooks et al. (2016) [23]	UK	Involved people with dementia as both advisers and participants in research about the use of life story work	Advisers	PwD	• Individual meetings	Advisers’ views on outcomes of life story work helped researchers with literature review, informed on ethical issues involved in doing focus groups with PwD, advised on information sheets and consent for PwD to ensure it’s clear and easy to understand, validated focus group findings from the first stage of the project. Informed on style of film for dissemination	Partnership between the research team and Innovations in Dementia to set up a network of advisers ensured that people with dementia were able to contribute in a real and valuable way to the research. However, the process of using consultees was lengthy and complex	NR	NR	NR
Design	Sheree et al. (2017) [24]	UK	Evaluate the design against stakeholder requirements: obtain feedback from PPI representatives and clinical experts on the usability of the adapted therapy	Patient and Public Involvement	Parkinson related dementia and caregivers	• PPI session	The final version of the therapy manual incorporated feedback from PPI session. PPI input agreed that the intervention was easy to engage in the session and to stimulate conversation using the topic materials, and that the discussion was stimulating and interesting. Also agreed that the session content and images were clear and easy to understand, that there was opportunity to personalize the session and that they would like to do a similar activity in the future.	NR	NR	NR	
Design	Alan et al. (2016) [25]	UK	NR	Patient and public involvement group	PwD and Carers	• Interviews	Obtained feedback on the acceptability and feasibility of the toolkit, this was used to modify the toolkits prior to piloting	NR	NR	NR	NR
Undertaking / Managing	Burnell et al. (2012) [26]	UK	To develop a peer support intervention for family carers of people with dementia by involving service users in research	Involving service users	PwD and caregivers	• Modified Delphi process combined with a consensus conference• Anonymous reader consultation	Intervention was renamed, the role of the carer supporter defined, information booklet less formal and presentation style changed	Importance of involving service users in the development of complex interventions in making the intervention and consent documents as appropriate and feasible as possible to the target recipients	Peer support among small reference group, virtual lay group accommodates members with mobility issues, PPI group as members of the steering committee – good practise involving carers with direct experience, involving carers face-to-face and virtually is invaluable in shaping a programme for the end user	NR	NR
Undertaking / Managing	Littlechild et al. (2015) [27]	UK	Older people with dementia were involved as co-researchers and worked with academic researchers in all stages of the research process, exploring other older people’s experiences of transitions between care services	Co-researchers	PwD	• Participatory approach	Co-researchers worked in all stages of the research process, in planning and carrying out the interviews, although the academic researchers were conscious of the power they held in terms of resources and responsibility for the outcomes, they invested considerable time and effort into promoting equality in their relationships with the co researchers	It is possible to involve older people with dementia in a meaningful way in research processes and that both co-researchers and participants can benefit significantly from their participation	NR	• Semi-structured interviews • Focus groups	Co-researchers perspectives:Participation in the study was to ‘make a difference’, and had some advantages of ‘telling things as they are’. Personal benefits of involvement referred to gaining knowledge, enhancing skills, developing networks and new opportunities for involvement. The project helped them to own and affirm publicly their dementia identity in a way that they felt benefitted others, challenging negative expectations of both self and othersStatutory organisations’ perspectives:The feeding back of findings via the reading of narrative experiences was regarded as both powerful and refreshingVoluntary organisations’ perspectives:Co-research approach had led to fuller and richer data. Involving co-researchers with dementia was seen as challenging negative stereotypes Academic researchers’ perspectivesCo-researchers were sometimes sensitive to different issues, were more adept at ‘tuning in’ to participants’ communications. However, there was a tension between the data generated through the interactions of co-researchers and participants in the interviews and the data required to answer the research questions
Undertaking / Managing	Mockford et al. (2016) [28]	UK	Setting up the research study with lay members as part of the research team	Co-researchers	PwD and carers	• Discussions	Developing ideas which are important to people living with memory loss, high levels of interest from volunteer groups. Organisational challenges were met in the requirement for research passports and with payment methods for the co-researchers. Training was beneficial but incurred extra costs for repeated training days	Overall the benefits outweighed the challenges which were overcome to varying degrees. Buy-in to service user involvement in research studies could be improved by clarifying the requirements for NHS Trust approval and by simplifying the system for financial reimbursement to lay co-researchers.	NR	NR	NR
Undertaking / Managing	Swarbrick et al. (2016) [29]	UK	Development of the CO-researcher INvolvement and Engagement in Dementia (COINED) Model, which was co-produced alongside three independent groups of people living with dementia	Co-researchers	PwD	• Participatory Action Research• Discussions	Group members developed a more comprehensive compendium of themes of co-researcher involvement and engagement model. Each component of the model represents inclusivity, mutual respect and empowerment which are at the very core of the Neighbourhoods and Dementia Study	Developed the co-researcher involvement and engagement in dementia (COINED) Model, and identified ways in which people living with dementia wish to be involved as co-researchers in the research process.	NR	NR	NR
Undertaking / Managing	Giebel et al. (2017) [30]	UK	To develop a dementia toolkit, synthesising the evidence for home support and involving patient and public throughout the programme	Public involvement	PwD, caregivers and public members	• Small reference group • Virtual lay advisory group	Feedback on the proposal, protocol and research design of the suggested programme, agreed proposed research, amendments made to interventions, contributed towards devising a model using specialist software, contributed to the evidence synthesis components, revision to patient pathway – economic model, amendment to participant information sheet, case vignettes agreed on	Virtual lay advisory group allows access for anyone regardless of location and is less time consuming. Involving carers and patients both face to-face and virtually to shape both the design and methods of data collection has proven an invaluable source of knowledge to adapt studies of the programme better to the real-life experience of patient and public	NR	NR	NR
Undertaking / Managing	Schipper et al. (2014) [31]	Netherlands	Patients with Parkinson’s disease were engaged to list priorities for research to complement the professionals’ research agenda	Engaged	PwP and a spouse of a patient with PwP	• Interviews • Focus groups • Questionnaire • Voiceover group meetings	Developed a shared research agenda, prioritised the research topics, research process, commented on preliminary findings	NR	NR	NR	NR
Undertaking / Managing	Morgan et al. (2018) [32]	UK	To collect Research Network volunteers and researchers perspectives to understand and demonstrate the real impact of the Research Network	Research Network volunteers	PwD and carers	• Lay reviewer• Monitor• Grant Advisory Panel lay member• Grant Advisory Board lay member• Research Strategy Council lay member• Research Network Area Coordinator	A series of detailed case studies, demonstrating the range of mechanisms through which impact is achieved on volunteers, on researchers, on research and on Alzheimer’s Society	PPI was seen as less influential in biomedical research than in care research. However, case studies demonstrate Research Network can contribute to communication,accessibility and research impact in biomedical research.	NR	• Online survey• Semi-structured interview or group interview	1. Impact on volunteers - Network volunteers share motivation to make a contribution to dementia research and, ultimately, to improve life for people affected by dementia. For several volunteers, involvement in the Research Network have led to further opportunities for involvement in dementia research2. Impact on researchers - Insight from volunteers helps to validate current ideas and inform the future focus of researchers work, and to focus on the long-term impact of their body of work. Improved researchers written lay communication skills and understand more about how people understand and interpret their work. This has particular benefits for early career researchers:3. Impact on research - Changing methodology, supporting ethics approval applications, ensuring that the outcomes of research produce useful benefits for people affected by dementia has led to change in research focus.4. Impact on Alzheimer’s Society - Ensured consensus, accountability and allowed Research Network to reach a consensus that is representative of the diverse views
Undertaking / Managing	Iliffe et al. (2013) [33]	UK	Describes the extent of PPI in research within the Dementias and Neurodegenerative Diseases Research Network (DeNDRoN), in terms of PPI activity at the different stages of the research cycle and within the different levels of the research network. Includes three case studies in which centrally managed PPI was called on to assist three research studies conducted within the network.	Patient and public involvement	Patients, carers and public	• PPI panels	• DOMINO‐AD study - PPI advised to promote the study more effectively in primary care and to make GP referrals of patients into the study easier, by informing practices of the study and inviting them to engage with the research team and identify potential participants. This had a positive impact, recruitment rates increased within the 2 sites after PPI consultation on recruitment issues, in comparison with all other DeNDRoN recruitment patterns for the DOMINO‐AD study.• MUSTARDD‐PD - PPI reviewers recommendation was that the word ‘dementia’ should be used less and be replaced by terminology which pertained to the range of potential symptoms and signs; the study team found many suggestions as valid and constructive and that they would be making changes to their documents as a result of the consultation process with the PPI reviewers.• RESULT - Members of the PPI reference panel contributed to designing a systematic review of literature and available data, reviews of GP records and hospital databases, and economic modelling of disease progression and cost at different stages. PPI representatives had a significant role in the final stage of the study. A questionnaire was developed with help of the patients and carers on the reference panel, and approved by an ethics committee, which allowed lay people to review the intended outcomes of studies, dissemination plans and implementation strategies.	More specific written guidance is needed to optimize the contribution of PPI in clinical research at all stages from design to implementation. Guidance should contain clear information about the level of support that is on offer for study teams, practical advice about PPI methods, and details of the resources available and advice on what resources will be needed	The three case studies exemplify how centrally managed PPI can contribute positively to clinical research, in different ways. The benefits of PPI in these studies were perceived positively by both research network staff and researchers, as they solved problems identified by the research teams themselves.	NR	NR
Analysing & interpreting	Bunn et al. (2015) [34]	UK	To test and contextualize the findings of a systematic review of qualitative studies looking at patient and carer experiences of diagnosis and treatment of dementia	Involving service users	Patient, public and professionals	• Focus groups • Semi-structured interviews	Findings from the focus groups and interviews were consistent with those from the systematic review	The involvement of service users and practitioners allowed a more nuanced understanding of the systematic review data	NR	NR	NR
Analysing & interpreting	Martin et al. (2015) [35]	UK	To give members of the public the opportunity to offer their perspectives and to comment on the findings of a systematic literature review looking at attitudes and preferences towards screening for dementia	Patient and public involvement Event	Public members and carers	• Patient and public involvement Event	23 key themes emerged. PPI exercise suggest the acceptability of screening is dependent upon a variety of factors including personal beliefs, experiences and attitudes to health	Inclusion of lay participants in the analysis and the writing of the article was helpful because it allowed for the research team to confirm their interpretation of the discussion	NR	NR	NR
Analysing & interpreting	Stevenson et al. (2017) [36]	UK	Involving individuals with dementia as co-researchers in a qualitative analysis	Co-researchers	PwD	• Sessions involved in deriving meaning from the data, identifying and connecting themes	The analysis generated additional sub-topics for exploration in subsequent interviews. The session also led to development of ideas for dissemination of findings.	Involving users with dementia in analysis impacted on the overall quality of the study by allowing for inclusion of multiple perspectives in interpretation of findings and generating new insights to be explored in further interviews	NR	• Open-ended questions in a paper format	Usefulness of the role-play exercises as an opportunity to ‘actually think about a situation that happened to someone else’. Benefitted from taking part in an activity that required them to reason, use their cognitive abilities, meet with others of similar ability and hear the opinions of other people with dementia.

*NR Not reported

### How PPI with people living with dementia is being carried out

The key areas of PPI were in identifying and prioritising research questions (*n* = 4), research design (*n* = 5), undertaking and managing research (*n* = 8), and in data analysis and interpretation (*n* = 3).

The terms used to refer to PPI varied widely including ‘priority setting partnership’ [17, 18], ‘Public Patient Involvement workshop’ [19], ‘user participation’ [20], ‘user involvement’ [21], ‘advisers’ [22], ‘co-researchers’ [23–25] and public ‘engagement’ [26].

Fourteen studies involved both patients, care partners and members of the public [17–19, 21, 26–35], five studies involved just patients [20, 22–25], and one study [36] involved only members of the public and care partners in the PPI activity.

A variety of terms were used to describe PPI activity, including workshops, drop-in sessions and meetings [27], individual meetings [22], modified Delphi process combined with a consensus conference and anonymous reader consultation [30], participatory approach [23], interviews, focus groups, questionnaire, voiceover group meetings [26] and a patient and public involvement event [36].

### The impact of PPI on people living with dementia, the public, dementia researchers, and the research process within the European Union

Only three studies formally evaluated the impact of PPI in dementia research [23, 25, 33]. Most studies reported impacts of PPI anecdotally. Of the three studies that included a formal evaluation of impact, Stevenson et al. [25] used a paper questionnaire asking what members liked most and least about the session, and also tried to capture their perspective on the benefits of being a co-researcher in the exercise. Littlechild et al. [23] used semi-structured interviews and focus groups to evaluate the impact of involvement in all stages of the research process from prioritisation and formulation of research questions, study design, recruitment, data analysis and interpretation to dissemination. Littlechild et al. [23] surveyed the viewpoints of co-researchers, statutory organisations, voluntary organisations and academic researchers [23]. Finally, Morgan et al. [33] employed an online survey, semi-structured interviews and focus groups to evaluate the impact of volunteers who provide PPI input in research projects funded by the UK Alzheimer’s Society. These studies identified the impacts of PPI in dementia research as follows:

Three studies described PPI in identifying and prioritising research questions [17, 18, 26]. Studies incorporating PPI in research design reported various impacts, particularly during development of non-pharmacological interventions for people living with dementia. For example Yates et al. [21] described how PPI informed development of a one-to-one, carer-led cognitive stimulation intervention for people living with dementia [21]. The authors [21] reported that PPI helped with the development of the drafts of the intervention manual and activity workbook in terms of language clarity and generation of ideas for the materials used in the intervention. Practical issues with the intervention were also identified by PPI feedback, including prioritising time to complete intervention sessions, and suggestions of ideas of how care partners can overcome barriers to completing the intervention. They also [21] reported that PPI feedback helped the research team understand reasons for non-adherence to the intervention and an awareness of the support that care partners may need in carrying out the intervention.

The following were identified as impacts of PPI input in undertaking and managing research: identifying issues of importance to patients and care partners during development of a research proposal [26, 32], assisting in the development of non-pharmacological interventions by making participant information sheets and consent documents appropriate for the target recipients [26, 30–32]. Interventions being re-named to make them sound more appealing and acceptable to potential participants, with a view to help with the recruitment rate for the intervention [30]. Two studies also involved PPI members in data collection as co-researchers [24, 32].

Studies that reported PPI in analysis and interpretation reported that PPI generated new insights and endorsed researchers’ interpretations of the findings [25, 35, 36]. For example, Stevenson et al. [25] described how researchers involved people living with dementia as co-researchers in analysis of quotations from qualitative interview data as part of a study on communication of health risks in dementia care.

### PPI group perspectives on review findings

As described earlier, we consulted a PPI group of people living with dementia and care partners to discuss the findings of the scoping review. They commented on the various PPI approaches identified in the review and felt that standardising PPI approaches might be useful to improve the quality of PPI in research. They felt a more standard approach to PPI could help people living with dementia understand what was expected in terms of their PPI role. The PPI group suggested that PPI approaches should consider the needs of participants, and should reflect on how best to involve particular participants to facilitate meaningful input. They emphasised that PPI input should be obtained at an early stage in the research process to ensure that patients and care partners are involved in research priority setting and identifying research outcomes that are relevant to them. The PPI group felt that it was important to be involved in all stages of research to ensure that the relevance of the research is maintained throughout, including the design of the study materials, facilitating participation in the research and identifying dissemination methods to reach relevant audiences. The group expressed a preference for PPI approaches that involve small group meetings to ensure that the discussion is focussed on key issues and also so that individuals could be more closely supported to provide input. With respect to the general lack of formal evaluation of the impact of PPI, the PPI group suggested that this could be due to researchers not thinking far enough ahead in planning PPI input to their research. They thought it was important that PPI should be acknowledged in published materials to underpin the value of PPI input in research.

## Discussion

### How PPI with people living with dementia is being carried out in dementia research in the European Union

To our knowledge, this is the first review to examine the types and impact of PPI in dementia research in the EU. There were increased reports of PPI activity in dementia research within the UK from 2012 onwards, although there were only a few published reports of PPI in dementia research from the EU. The range of terms used to refer to PPI by authors in the selected studies in this review was varied. The terminology used to refer to PPI may be based on different understandings and different objectives for PPI in each study. Use of different terminology for PPI may also reflect debate about the meaning and types of PPI appropriate for different research contexts [37–39]. The studies identified in this review also used various different approaches to PPI (see Table 4). Approaches may vary according to the aims of the specific PPI. Conversely, the various PPI approaches posed challenges for this review in terms of (i) systematically describing PPI approaches and (ii) identifying which approaches are effective or appropriate for particular research objectives. Despite variation in terminology and approaches, all of the PPI reported in this review related to research involvement ‘with’ or ‘by’ members of the public rather than ‘to’, ‘about’ or ‘for’ them [1]. The key principal of working ‘with’ or work ‘by’ members of the public [1] may serve as a starting point for future PPI in dementia research. Anecdotal evidence reported in this review suggests that a variety of PPI approaches are effective in facilitating involvement and impact on research. PPI is a rapidly developing area, and a variety of PPI approaches is likely to be appropriate due to variation in research study context, patient population and research objectives [5, 40–43]. Previous suggestions [44–47] for effective PPI included utilising PPI from the early planning stages of research by assisting researchers to identify different research topics, to adapt their research questions and subsequently also helps researchers design and conduct their research in a way that potential participants deem to be ethically satisfactory.

PPI input was reported at all stages of research, including the initial proposal for funding [31], design [20–22, 28, 29] and data analysis and interpretation [25, 36]. Whilst most of the studies incorporated PPI input into at least one single stage of the research process, 8 studies used PPI in all stages of undertaking and managing the research. Theoretically, PPI could occur at all stages of research. However there are challenges around maintaining long term involvement of members of the public in research projects [32]. The challenges in maintaining the involvement of people with progressive medical conditions such as dementia are particularly acute. Giebel et al. [32] suggested on-going recruitment of PPI representatives to ensure continued PPI in long term research projects.

### The impacts of PPI on people living with dementia and the public, dementia researchers, and the research process within the European Union

PPI is suggested to increase the cost effectiveness of research by ensuring that research outputs are appropriate to the patient group of interest [48]. In this review, just three studies included a formal evaluation of the impact of PPI, with 17 studies reporting anecdotal impacts of PPI. The reported impacts of PPI depended on the objective of the PPI and stage of research that PPI was conducted (e.g. in setting research priorities versus research design).

The lack of formal evaluation and the different approaches that were used in studies that did attempt to formally evaluate the impact of PPI made it difficult to establish whether one approach may be more effective than another, or even whether PPI in dementia research does deliver consistent benefits. Other reviews of PPI in health research have previously identified a lack of evidence for the impact and benefits of PPI, and where studies have reported the impact of PPI, the quality of the evidence is low [2, 5, 49].

A review by Boote et al. [50] identified that inadequate resources are allocated for monitoring and evaluation of PPI impact and PPI impacts tend not to be systematically recorded during the process of PPI. There is a need to substantially improve the evidence for the impact and cost effectiveness of PPI [51, 52].

#### Strengths and limitations of the review

As only abstracts written in English were included, some relevant EU studies may have been missed. PPI activities may be under-reported in general [23, 25], so some relevant PPI activities in the EU may not have been identified in this review. With the exception of the British Medical Journal (BMJ), most journals do not request information about PPI, so PPI activities may go unreported. Although BMJ have introduced the requirements for specific information about PPI, a study [53] found that only 11% of studies actually report PPI activity. No studies in this review used a standardised format for reporting PPI such as the GRIPP [15, 54] which may have reduced the quality of reports of PPI. Lack of standards for reporting PPI made it difficult to extract relevant information from the papers.

A strength of the review was the involvement of a PPI group in interpreting the review findings. A very wide range of search terms was used to capture variation in terminology used to describe PPI. A large section of databases, including those indexing grey literature were systematically searched.

### Future challenges and recommendations

Methodology and terminology used to refer to PPI varied. In different contexts, different words are used for similar activities, or the same terms were used interchangeably to refer to different things. A lack of standard terminology for PPI was identified in previous reviews of PPI [5, 39]. Telford, Boote and Cooper [49] recommended that detailed accounts of PPI should be included in research reports and publications. Subsequently, substantial work has been undertaken to develop reporting standards for PPI in the form of the GRIPP [54], and in turn the shorter, revised GRIPP2 [15]. More robust reporting of PPI could guide future research in PPI, develop best practice for PPI [55] and reduce research waste via reducing ineffective application of PPI [56, 57].

Perhaps related to the issues of varying terminology and approaches, a key shortcoming identified in this review was the lack of good quality evidence for the impacts of PPI in dementia research. A better understanding of the benefits and impact of PPI in dementia research would encourage researchers to embed PPI within research culture and provide an incentive for patients and members of the public to be involved. As good practise, effective PPI could be appropriately costed in proportion to the overall budget. PPI costs include staff time and expenses, PPI members’ costs and expenses, administration, training for staff and PPI members, transportation, venue hire, and monitoring and evaluation costs [2, 58]. It is critical that there be good quality evidence for the benefits and impacts of PPI in dementia research in order to convince researchers and funders for the need to appropriately fund PPI activities. Evidence of impact of PPI is not the number of patients involved in PPI, but should relate to the useful difference the PPI made to the research. Establishing an evidence base for PPI in dementia research requires formal evaluation of relevant impacts and systematic reporting of PPI [41, 43, 54]. Although some guidance for reporting of PPI is available [15, 59–61], the GRIPP 2 [15] reporting checklist provides one possible framework for describing PPI contributions to research. The GRIPP 2 [15] checklist has a particular emphasis on describing PPI activities. But a shortcoming is that the GRIPP2 has no standards for evaluating and reporting the quality of the PPI or for systematically quantifying impact of PPI on research. In the present study, we adapted the GRIPP2 by adding two sections to describe the method of evaluation of the impact of PPI (if included) and what the reported impacts of PPI were. Future iterations of the GRIPP2 should include evaluation of PPI impact.

The great majority of the studies identified in this review were carried out in the UK. Lessons learned in UK could inform methodological development of PPI in dementia research in EU. However, with Brexit there remains uncertainty about the UK’s capacity to engage and collaborate in future research in EU, in addition to challenges posed by more restricted mobility of health care workers and researchers between the UK and the rest of EU. These challenges may be addressed by accelerating the development of the European Research Area [62], including reducing barriers to movement, a shared UK and EU research agenda, and development of European policies to overcome exclusion and promote participation of people living with dementia in research. UK researchers must persevere and continue to contribute to the discourse concerning PPI in dementia research in EU.

European charity and governmental research policies are beginning to advocate inclusion of PPI in dementia research [10–13]. PPI in dementia research may be further facilitated if peer reviewed journals, sponsors and funding institutions were to require PPI. The British Medical Journal (BMJ) released a 2014 guide for authors to report PPI in research published in the BMJ [63]. If no PPI activity took place, authors are expected to clearly report this. Adoption of similar guidelines by dementia research funders and peer reviewed journals may promote PPI in dementia research.

## Conclusion

This review describes the various approaches to as well as the strengths, weaknesses and opportunities for PPI in dementia research in EU. The number of published studies reporting PPI in dementia research is growing, reflecting recognition of the importance and the feasibility of PPI in dementia research. A variety of PPI methodologies were used at all stages of the research process, all of which may be appropriate in different contexts according to the research question and aim to be addressed, the characteristics of PPI members and the resources available.

Variation in the terminology used to describe PPI and variation in the quality of reporting limited our capacity to interpret and synthesise the research. Due to the lack of a standard definition of PPI, there is a need to identify universal principles of involvement that can be implemented in dementia research. PPI in dementia research should be evaluated in relation to both the effectiveness of implementation and in accomplishing its objectives in informing research. Evaluation of PPI should be based on good quality research designs with rigorous standards of reporting in order to justify the effort and cost associated with PPI in terms of benefits to researchers, patients and the general public.

## Additional files

Additional file 1: PRISMA Extension for Scoping Reviews (PRISMA-ScR): Checklist Guidance and checklist for PRISMA Extension for Scoping Reviews (PRISMA-ScR): Checklist and Explanation. (DOCX 105 kb)

Additional file 2: Full Search Terms Full search terms used in database searches. (DOCX 12 kb)

Additional file 3: Data extraction form Data extraction form used. (DOCX 11 kb)

## Data Availability

The data generated or analysed during this study are included in this published article (and its supplementary information files).

## Abbreviations

EU: European Union
GRIPP: Guidance for Reporting Involvement of Patients and the Public
PPI: Patient and Public involvement
