# Supplementary material for: Patient and public involvement in dementia research in the European Union: a scoping review
Source: BMC Geriatr. 2019 Aug 14;19:220. doi: 10.1186/s12877-019-1217-9 (PMC6694462; doi:10.1186/s12877-019-1217-9)
Supplement: Supplementary file 2 — Full Search Terms Full search terms used in database searches. (DOCX 12 kb) [file 12877_2019_1217_MOESM2_ESM.docx]

Search terms for Ovid and PubMed:

1. Dementia (Mesh) **adj5** 14, 15, 16, 17, 18, 19, 20, 21, 22, 23, 24, 25, 26, 27
2. Dementia with Lewy bod*(Mesh) **adj5** 14, 15, 16, 17, 18, 19, 20, 21, 22, 23, 24, 25, 26, 27
3. Lewy bod* (Mesh) **adj5** 14, 15, 16, 17, 18, 19, 20, 21, 22, 23, 24, 25, 26, 27
4. Mild cognitive impairment **adj5** 14, 15, 16, 17, 18, 19, 20, 21, 22, 23, 24, 25, 26, 27
5. MCI **adj5** 14, 15, 16, 17, 18, 19, 20, 21, 22, 23, 24, 25, 26, 27
6. AD **adj5** 14, 15, 16, 17, 18, 19, 20, 21, 22, 23, 24, 25, 26, 27
7. Alzheimer* disease (Mesh) **adj5** 14, 15, 16, 17, 18, 19, 20, 21, 22, 23, 24, 25, 26, 27
8. Memory loss (Mesh) **adj5** 14, 15, 16, 17, 18, 19, 20, 21, 22, 23, 24, 25, 26, 27
9. Huntington* disease (Mesh) **adj5** 14, 15, 16, 17, 18, 19, 20, 21, 22, 23, 24, 25, 26, 27
10. Primary progressive aphasia (Mesh) **adj5** 14, 15, 16, 17, 18, 19, 20, 21, 22, 23, 24, 25, 26, 27
11. Vascular dementia (Mesh), Parkinson* disease (Mesh) **adj5** 14, 15, 16, 17, 18, 19, 20, 21, 22, 23, 24, 25, 26, 27
12. Frontotemporal dementia (Mesh) **adj5** 14, 15, 16, 17, 18, 19, 20, 21, 22, 23, 24, 25, 26, 27
13. Frontotemporal Lobar degeneration (Mesh) **adj5** 14, 15, 16, 17, 18, 19, 20, 21, 22, 23, 24, 25, 26, 27
14. Patient* involvement,
15. Public involvement,
16. Patient* and public Involvement,
17. Involving patient*s,
18. User led,
19. Service user involvement,
20. Patient* participation,
21. Patient* and public voice,
22. Study partner,
23. Participatory research,
24. Consumer involvement,
25. Citizen participation,
26. Patient* and service user involvement,
27. User involvement
