# Supplementary material for: Patient and public involvement in dementia research in the European Union: a scoping review
Source: BMC Geriatr. 2019 Aug 14;19:220. doi: 10.1186/s12877-019-1217-9 (PMC6694462; doi:10.1186/s12877-019-1217-9)
Supplement: Supplementary file 3 — Data extraction form Data extraction form used. (DOCX 11 kb) [file 12877_2019_1217_MOESM3_ESM.docx]

**Data Extraction Form**:

| Title - Journal |  | Additional notes: |
| --- | --- | --- |
| Author |  |  |
| Country |  |  |
| Year of publication |  |  |
| Aim/s of the study |  |  |
| Study design |  |  |
| Study population |  |  |
| PPI term used |  |  |
| Methods used for PPI |  |  |
| Was PPI impact evaluated |  |  |
| If yes - methods used for evaluation |  |  |
| Findings from the evaluation. |  |  |
